# Periapical healing outcome following single visit endodontic treatment in patients with type 2 diabetes mellitus

**DOI:** 10.4317/jced.52859

**Published:** 2016-12-01

**Authors:** Sandeep Rudranaik, Moksha Nayak, Medha Babshet

**Affiliations:** 1 MDS, Reader, Department of conservative dentistry & endodontics, Sri Hasanamba Dental College and Hospital, Hassan, India; 2 MDS, Principal, Department of conservative dentistry & endodontics, KVG Dental College and Hospital, Sullia, India; 3 MDS, Reader, Department of oral medicine and radiology, Sri Hasanamba Dental College and Hospital, Hassan, India

## Abstract

**Background:**

The prevalence of apical periodontitis in diabetes mellitus patients is high. The altered immunity in diabetes affects the healing process of periapical tissue. Single visit endodontic treatment has shown to increase the periapical healing rate with better patient compliance. Hence the present study aims at evaluating the clinical and radiographic healing outcome of single visit endodontic treatment, in type 2 diabetes mellitus patients with periapical disease.

**Material and Methods:**

Eighty patients with periapical disease were divided into 2 groups of 40 each: Group I, Control subjects and Group II, Type 2 diabetics. Glycosylated hemoglobin levels were assessed preoperatively and at follow up intervals in diabetics. Pre-operative assessment of periapical status was done using CPDR (Clinical periapical diagnosis of root), QLDR (Qualitative radiographic diagnosis of tooth) and QTDR (Quantitative radiographic diagnosis of tooth) criteria. Postoperative healing was evaluated following single-visit endodontic treatment by Strindberg criteria.

**Results:**

Group 2 subjects had chronic and exacerbating lesions with significantly larger lesions (*p*=0.029). 100 % clinical healing outcome in diabetic group was seen in two months. Group 2 showed 85% success in one year on radiographic evaluation. Poor controlled diabetics showed failure compared to fair and good controlled.

**Conclusions:**

Type 2 diabetics had chronic and larger sized lesions when compared to control subjects. The periapical lesions in patients with poor diabetic control showed failure. The clinical and radiographic healing outcome of single visit endodontic therapy was delayed in diabetic patients.

** Key words:**Apical periodontitis, diabetes mellitus type 2, endodontics, periapical lesion, strindberg criteria.

## Introduction

Diabetes mellitus is a common metabolic disorder characterized by various phenotypes of hyperglycemia. It is broadly categorized into Type 1 and Type 2 diabetes mellitus, among which type 2 is the commonest. The alteration in neutrophil function and failure to deliver the humoral and cellular components of the immune system in diabetic patients increases the risk of episodes of infection (1). Diabetes mellitus has its effects on pulp and periapical diseases and their treatment outcome (2). The prevalence of periapical infection is greater in diabetics in comparison to non-diabetics (3). The prevalence of apical periodontitis in type 2 diabetics is found to be 81.3% (4). Similar finding was observed by Marroto *et al.* and Lopez *et al.* in their research projects (5,6).

The tooth with apical periodontitis is managed by root canal therapy. The success rate of root canal treatment has been generally regarded as 87% (7). The scientifically documented procedure for the best results in canal disinfection is based on complete debridement, irrigation and obturation of root canal in single visit, reducing the risk of inter-appointment infection (8). Single visit root canal treatment has several advantages such as reduced flare-up rates (9), good patient acceptance and practice management, and is more effective in teeth with apical periodontitis with 6.3% higher healing rate than multiple visits (10).

Hence the present study was performed to evaluate both clinically and radiographically the periapical condition of the teeth in type 2 diabetic patients and thereby assess the healing outcome of single visit endodontic treatment in them.

## Material and Methods

80 subjects aged 20-60 years with irreversible pulpitis and apical periodontitis, requiring endodontic therapy were selected and divided into 2 groups.

-Group I - Control group: 40 normal subjects with no history of systemic disease and normal glycosylated hemoglobin levels (HbA1c >6.5 <7.5).

-Group II- Diabetic group: 40 subjects with diabetes mellitus, diagnosed by glycosylated hemoglobin > 7.5%, without any habits and other systemic diseases.

The approval was obtained from the institutional ethical committee to conduct the study and informed consent was taken from all the subjects. Diagnosis of apical periodontitis was done by clinical and radiographic examination. Pre-operative assessment of periapical status which included pain, apical tenderness & sinus tract was done using CPDR (Clinical periapical diagnosis of root), QLDR (Qualitative radiographic diagnosis of tooth) and QTDR (Quantitative radiographic diagnosis of tooth) criteria (11). The method of viewing the radiograph was standardized. Single-rooted teeth were diagnosed according to these criteria and in multi rooted teeth, most severely affected root was considered.

In group II Glycosylated Hb levels were assessed both preoperatively and at follow up intervals postoperatively. Preoperative, post-obturation, and follow-up radiographs were made in a 70 kVp, 10 mA IOPAR machine using paralleling technique. Standardized processing technique was used in order to obtain optimal diagnostic quality of the radiographs.

-Procedure for single sitting root canal therapy 

The selected tooth was anesthetized with 2% Lignocaine. Single visit endodontic treatment was carried out with strict aseptic measures. Working length was confirmed and adjusted as needed by using straight and angled radiographs using Ingle’s method. The root canals were instrumented in a crown down technique (12) with protaper rotary NiTi file (Dentsply) and RC Prep; canals were irrigated with sodium hypochlorite & chlorhexidine. Protaper gutta-percha master cone was selected corresponding to that of last protaper finishing file (13) used & confirmatory IOPAR and RVG were taken. The obturation was completed with zinc oxide eugenol sealer.

Postoperative evaluation of healing was done at first week, second week, first month, second month, sixth month and after one year, using Strindberg criteria (14) The results were statistically evaluated using Student unpaired T test, Chi-Square test and Freidman’s test.

## Results

The mean age of Group I patients was 34 yrs and Group II was 44 yrs. Unpaired t test showed no significant difference between the groups (*p*=0.087). In Group I, 24 were males and 16 were females and Group II 18 were males and 22 were females. Chi-square test showed no significant difference between groups.

On preoperative assessment of glycosylated hemoglobin patients with HbA1c >6.5 <7.5 were included in Group I. Among Group II patients six were poor controlled [≥9.5%] diabetics, 26 were fair [8.5-9.5%] and eight had good control [7.5-8.5%].

The preoperative assessment of pain, sinus tract and apical tenderness using Friedman’s test showed statistically significant improvement in both groups over a period of 6 months ( Table 1).

**Table 1:**
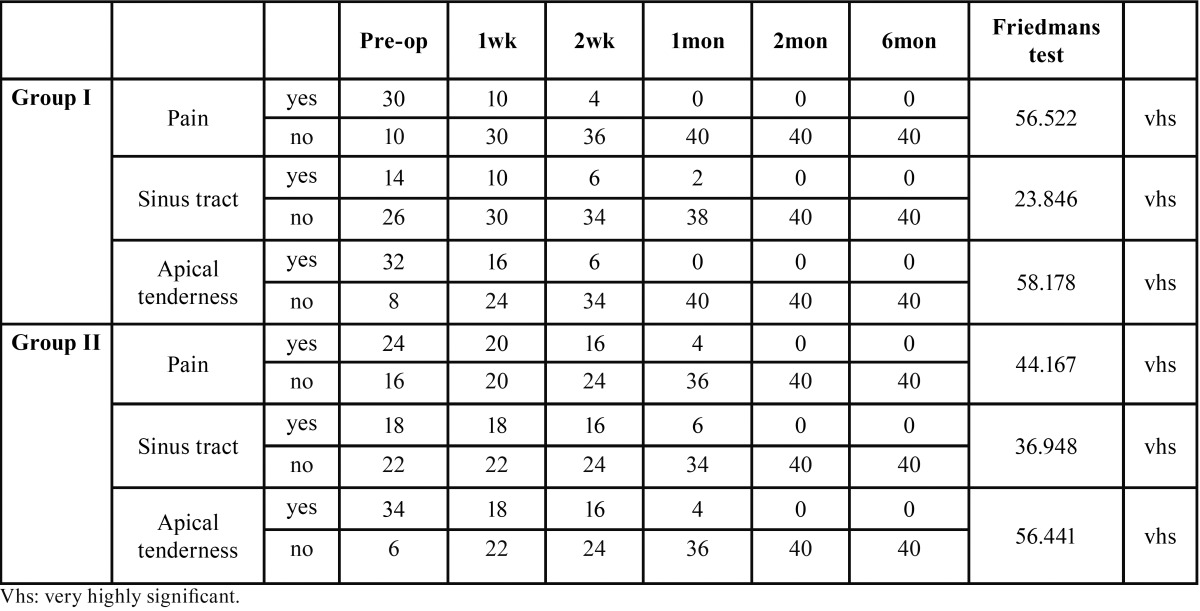
Healing outcome of teeth with Preoperative pain, sinus tract and apical tenderness using Friedman’s test.

Based CPDR and QTDR criteria, the acute lesions were more in group I, whereas chronic lesions were more in group II ( Table 2). Preoperative assessment of periapical status according to, QLDR Criteria (Qualitative radiographic diagnosis of tooth showed increased occurrence of periapical disease in group II ( Table 3). The group II subjects had significantly larger sized lesions compared to those in group I ( Table 4).

**Table 2:**
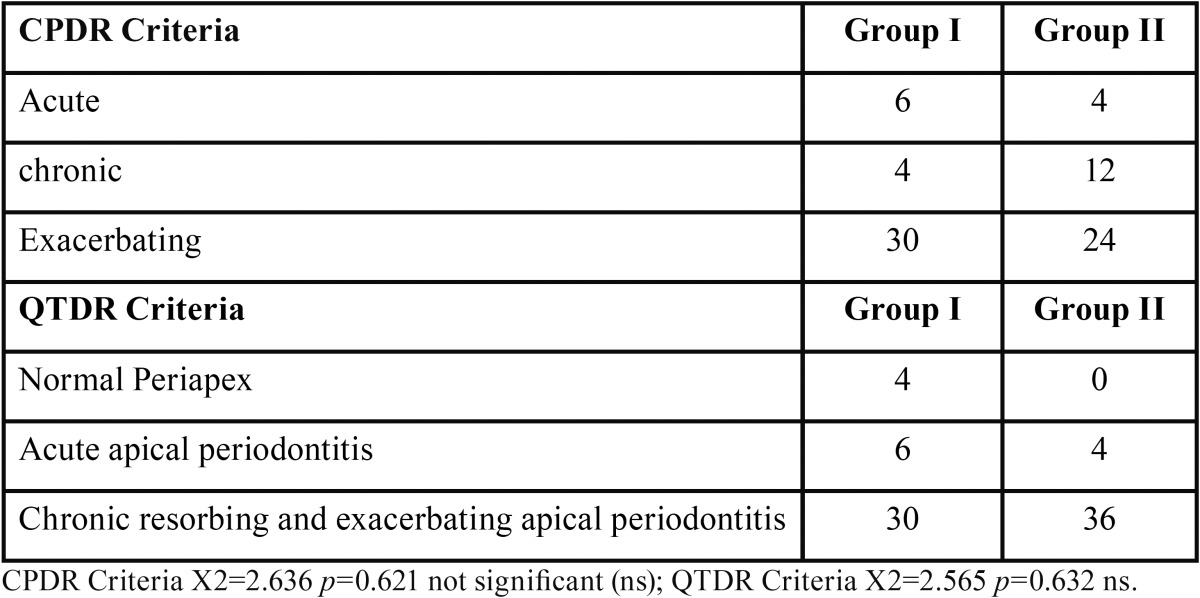
Preoperative assessment of periapical status according to, CPDR Criteria and QTDR.

**Table 3:**
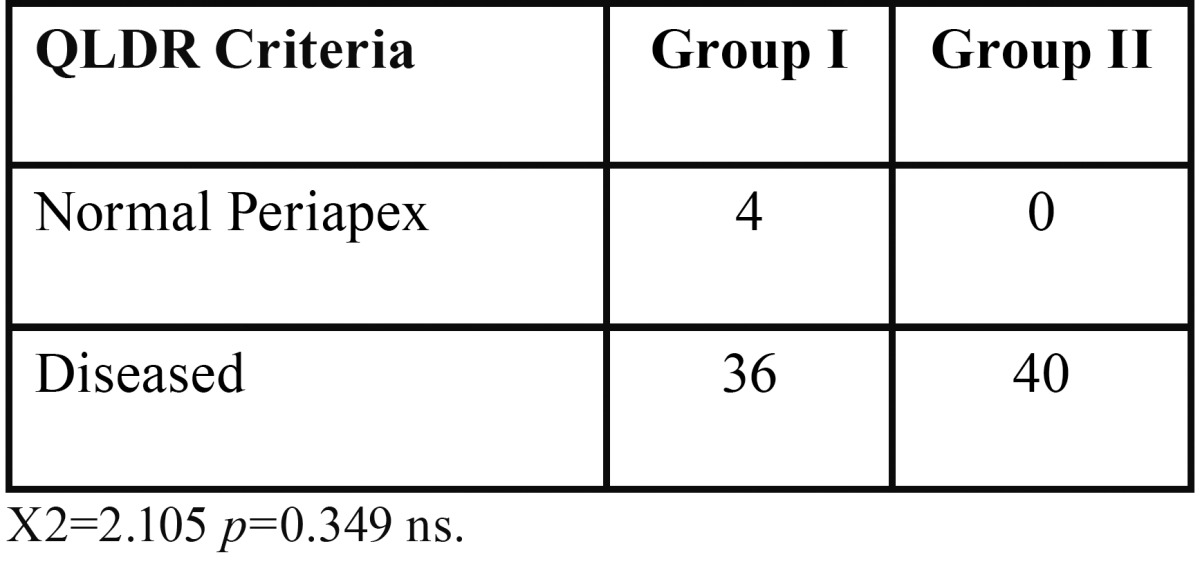
Preoperative assessment of periapical status according to, QLDR Criteria.

**Table 4:**
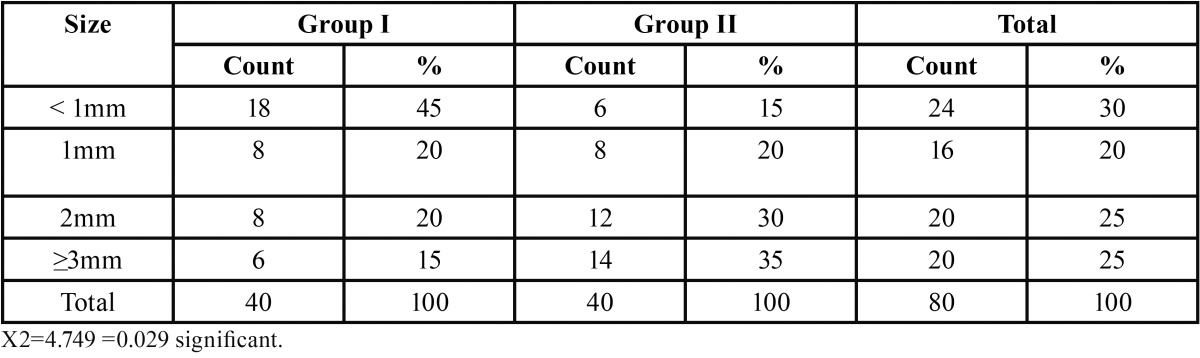
Preoperative size of periapical lesion in all subject groups.

A significant difference was observed in the size of periapical radiolucency from first month to one year. The lesions ≤1 mm healed within 6 months, whereas only few teeth with large periapical lesions healed completely by one year of evaluation. Higher failure rate was seen in larger sized lesions ( Table 5).

**Table 5:**
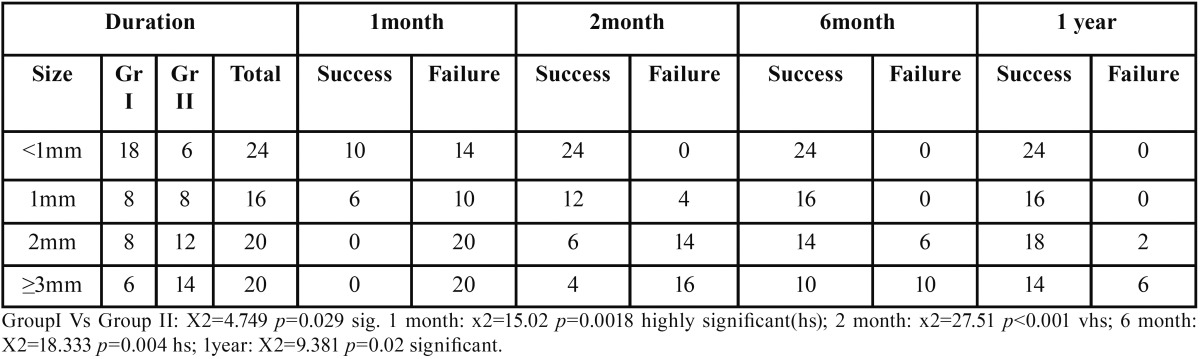
Relation between preoperative size of periapical radiolucency and treatment outcome.

The clinical evaluation of periapical healing outcome using Strindberg criteria showed 100% success in group I within 1 month, however it was observed after 2 months in Group II ( Table 6).

**Table 6:**
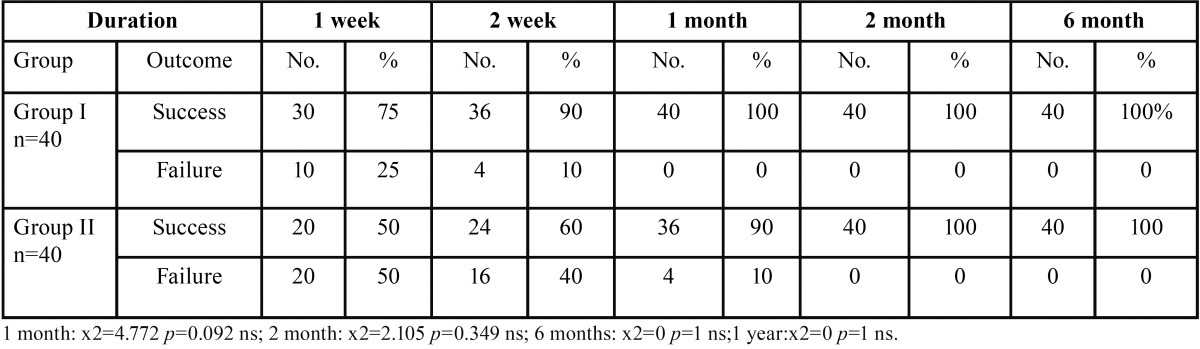
Evaluation of periapical healing outcome clinically over a period of 6 months: (adopted from Strindberg).

The radiographic evaluation of periapical healing outcome over a period of 1 year using Strindberg criteria showed significantly (*p*= 0.0026) low success rate in the first month which improved to 85% over a period of one year. However a success rate of 100% was observed in group I in 6 months ( Table 7).

**Table 7:**
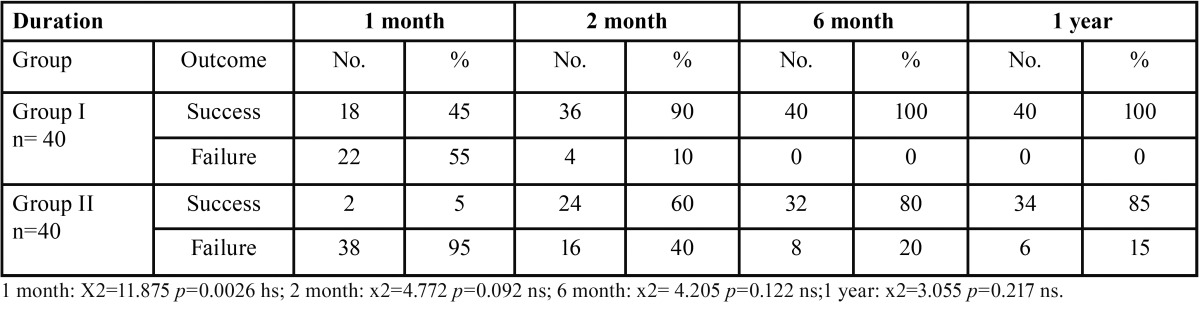
Evaluation of periapical healing outcome radiographically over a period of 1 Year: (adopted from Strindberg.

On one year follow up, six patients had poor controlled diabetes, 20 showed fair control and 14 had good control. Clinically healing was observed in all diabetic patients; and radiographically, all patients except the poor controlled diabetic patients showed successful healing. Mean HbA1c % preoperatively was 8.6800 and after one year it was 8.3300 with a mean difference of 0.3500. No statistical difference was seen between preoperative evaluation and after one year of evaluation of HbA1c %.

## Discussion

In the present study the average age of patients ranged from 30-40 yrs with no statistically significant difference. Moreover gender differences were also not significant. Irreversible pulpitis with apical periodontitis is the most common infection of the root canal system. The prevalence of apical periodontitis increases with age and it may rise to 62% in individuals over 60 years (7).

In the group II, majority of patients had fairly controlled diabetes. In the present study the preoperative HbA1c levels ranged from 7.8 to 10.4% with a history of diabetics ranging from 3-11 years.

The periapical status of each tooth in the present study was evaluated according to the 3 indices: (i) The clinical periapical diagnosis of root (CPDR), based on the diagnosis of the pulp and the root with the most extensive periapical involvement; (ii) Qualitative radiographic diagnosis of tooth (QLDR), the actual radiographic status of each individual root apex and differentiates between radiographically normal and diseased periapex; and (iii) Quantitative radiographic diagnosis of tooth (QTDR), the extent of periapical pathosis in millimeters (11).

-Preoperative assessment

In the present study pain was more common in Group I, whereas presence of sinus tract and apical tenderness were frequent in diabetic group ( Table 1). As per CPDR and QTDL criteria, in the present study, chronic and exacerbating lesions were seen more in type 2 diabetics ( Table 2). Falk, Hugoson and Thorstensson found similar correlation, who demonstrated long duration diabetics exhibit teeth with greater extent of periapical lesions than non diabetics (15). Elfving *et al.*, found diabetics with poor glycemic control have a higher rate of asymptomatic tooth infection (16).

Qualitative assessment using QLTR criteria showed more number of teeth with diseased periapex in diabetics than control ( Table 3). The compromised circulation within pulp due to endarteritis obliterans and lack of collateral circulation along with altered polymorphonuclear activity in diabetics are considered to result in an increased risk for infection or pulp necrosis (17). 

Uncontrolled diabetes has been hypothesized to have statistically significant association with apical periodontitis (4). The group II subjects had significantly larger sized lesions compared to those in group I ( Table 4). The results are in accordance with the study conducted by Iwama *et al.*, which showed that larger periradicular lesions were more and alveolar bone resorption was most severe in diabetics (18).

-Post-treatment Healing outcome

The endodontic treatment of infected teeth aims at elimination of bacteria from the root canal, thereby providing a favourable environment for healing. Several clinical studies report a success rate of endodontic therapy ranging from 87.4% to 94.5% (19,20). Single-visit RCT has been recommended for use in cases with pulpal inflammation, traumatic pulpal exposure, and necrotic pulp with a sinus tract (21,22).

In our study standard endodontic treatment protocol (23) was implemented in terms of root canal isolation, preparation and obturation. We used the new Progressively Tapered (ProTaper) NiTi rotary files for preparation of root canals. These files represent a revolutionary progression in root canal preparation procedures as they provide superior flexibility, unmatched efficiency and greater safety (13).

It has been hypothesized that pulpal necrosis in diabetics harbor a more virulent microbial profile, with significant increase in *Prevotella intermedia, Porphyromonas gingivalis, F. nucleatum, P. micros & Streptococcus* (23). Hence diabetics should be treated with effective antimicrobial root canal regimens. Chlorhexidine and sodium hypochlorite (NaOCl) have been shown to be the most effective root canal irrigants (24). In this regard, in our study these irrigants were used to irrigate the root canals.

The history of pain, presence of sinus tract and apical tenderness play a significant role in the outcome of endodontic treatment. The assessment of pain, sinus tract and apical tenderness using Friedman’s test showed very highly significant healing outcome over a period of 6 months ( Table 1). The success rate of teeth with preoperative sinus tract was less compared to teeth with no preoperative sinus tract. Our results were in agreement with the study performed by Chugal *et al.*, who found that success rate of teeth with a preoperative sinus tract was only 37.5% compared with a 62.7% success for teeth without sinus tract (11). The presence of sinus tract and apical tenderness was more common in Group II. The resolution of pain and apical tenderness in Group I was seen in one month, however in group II resolution of signs and symptoms took two months.

In the present study the smaller lesions showed successful healing outcome within six months, however the success rate of 70% ( 14 out of 20 patients) over a period of one year was observed in ≥3mm sized lesions ( Table 5). Friedman *et al.* in their review found that the success rate of teeth with apical periodontitis after initial treatment or retreatment was 74% to 86% (25). In the review, the follow up studies had varied periods of assessment ranging from six months to ten years. Bystrom *et al.* in their study suggested that as long as there is continuous decrease in the size of the lesion, the lesion need not be labeled as failure (26). Hence in our study, if the follow up period was more we would have expected higher success rates.

Clinical evaluation of periapical healing outcome over a period of six months using Strindberg criteria showed no statistical significance among both the groups over a period of six months. 100% success was observed in group I within 1 month, however it was observed after 2 months in Group II ( Table 6).

On radiographic evaluation a success rate of 90% in Group I and 60% in Group II were attained in 2 months. 15 % of Group II showed failure even after one year follow up, wherein 100 % of Group I had already achieved success in six months. Diabetic subjects with good and fair glucose control showed better healing than poor controlled diabetic subjects. Cheraskin and Ringsdort showed radiographic healing of periapical lesion following root canal treatment in a low glucose group were reduced by an average of 74% compared with a reduction of only 48% for a high glucose group (27). The lower success rates in diabetic patients could probably be due to impaired healing capacity and increased susceptibility to infection (28,29).

## Conclusions

The present study showed that patients with diabetes mellitus have more complex and compromised presentation of periapical disease and the condition poses a significant effect on the healing outcome of single visit endodontic treatment. The diabetic patients were more prone for chronic periapical disease with larger lesions. The periapical lesions were more prevalent in diabetic patients than in non diabetics. Smaller sized lesions healed faster whereas larger sized lesions showed higher failure rate. Healing outcome at the end of one year was poor in poor controlled diabetics when compared to fair and good controlled diabetics in group II. The clinical and radiographic healing outcome of single visit endodontic therapy was delayed in diabetic patients. Although few lesions still persisted over a period of one year radiographically, long term follow up would probably have shown further decrease in lesion size, adding to the success rate. Hence long term follow up studies are required to assess periapical healing and to determine effective treatment outcome.
